# Supercooled water escaping from metastability

**DOI:** 10.1038/srep07230

**Published:** 2014-11-27

**Authors:** Francesco Aliotta, Paolo V. Giaquinta, Rosina C. Ponterio, Santi Prestipino, Franz Saija, Gabriele Salvato, Cirino Vasi

**Affiliations:** 1 CNR-IPCF, Viale Ferdinando Stagno d'Alcontres 37, 98158 Messina, Italy; 2 Università degli Studi di Messina, Dipartimento di Fisica e di Scienze della Terra, Contrada Papardo, 98166 Messina, Italy

## Abstract

The return of supercooled water to a stable equilibrium condition is an irreversible process which, in large enough samples, takes place adiabatically. We investigated this phenomenon in water by fast imaging techniques. As water freezes, large energy and density fluctuations promote the spatial coexistence of solid and liquid phases at different temperatures. Upon synchronously monitoring the time evolution of the local temperature, we observed a sharp dynamic transition between a fast and a slow decay regime at about 266.6 K. We construe the observed phenomenon in terms of the temperature dependence of heat transfers from solid and liquid volumes already at their bulk coexistence temperature towards adjacent still supercooled liquid regions. These findings can be justified by observing that convective motions induced by thermal gradients in a supercooled liquid near coexistence are rapidly suppressed as the nucleated solid fraction overcomes, at low enough temperatures, a characteristic percolation threshold.

Water exhibits several pronounced thermodynamic, structural and dynamic anomalies^1^,^2^,^3^,^4^,^5^,^6^,^7^. In the last few decades, much attention has been devoted to explaining the apparent divergence of several transport properties of supercooled water below the homogeneous nucleation temperature^8^. This behavior has often been taken as an indirect evidence of a liquid-liquid phase transition hidden in the deeply supercooled regime^9^,^10^,^11^. Indeed, a first-order phase transition between a low-density liquid (LDL) and a high-density liquid (HDL) phase has been observed in the ST2 model of water^9^. A few indirect experimental indications on a liquid-liquid transition have been reported in water^12^,^13^ as well as in other molecular liquids^14^,^15^,^16^. However, a conventional (i.e., non transient) HDL-LDL phase coexistence would be likely inaccessible to any experimental investigation performed on bulk water because of fast ice nucleation at temperatures higher than those expected for the transition to occur.

The origin of water anomalies in the supercooled regime remains rather controversial^17^,^18^,^19^,^20^,^21^. In this respect, it is somewhat surprising that relatively few efforts have been made to clarify the process of water crystallization in the bulk and the kinetic pathways leading to ice nucleation (in this respect, notable exceptions are Refs. 22, 23). Liquid water is a strongly correlated molecular system whose microscopic and macroscopic behavior is dominated by intermolecular hydrogen bonds, which produce an unusually slow relaxation and a very rich phase diagram at low temperatures. This makes the description of ice nucleation so complicated that the size and structure of critical nuclei have not been safely ascertained yet. Certainly, a satisfactory description of ice nucleation goes well beyond the scope of the classical nucleation theory^8^,^24^,^25^. It has also been proposed that the polymorphism of supercooled water is relevant to ice nucleation since it may offer several alternative routes to the escape of the liquid from metastability^26^,^27^.

Recently, it has been argued that the widely adopted scheme describing the nucleation of a solid phase from a supercooled liquid as an isothermal process can be fundamentally wrong since it disregards any enthalpy contribution^28^,^29^,^30^. In fact, solid nucleation always occurs exothermically on a local scale, which implies that the liquid warms up while (partially) solidifying^31^. Generally speaking, any metastable system will eventually move in an irreversible way towards a stable thermodynamic condition, which means that the phase transformation does not require an energy exchange with the environment. In a globally isolated system some spontaneous fluctuations sooner or later will drive the system to the boundary between the metastable and the stable equilibrium basins in phase space and, if the amplitude of the fluctuation is large enough, the system will overcome the free-energy barrier between the two basins. Crossing the nucleation barrier is an irreversible process which takes place adiabatically in an isolated system (see, e.g., Ref. 32). A comparison between the enthalpy content of homogeneous supercooled water and that of its corresponding heterogeneous stable state at the melting temperature shows that the adiabatic description is indeed well suited for any experiment performed at ambient pressure (see the Supplementary Information). In an adiabatic setup the mole fraction of ice produced by the decay of a metastable fluid supercooled down to a given temperature *T* is^29^,^32^: 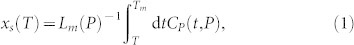
where *C_P_* is the isobaric heat capacity of the liquid, *L_m_*(*P*) is the latent heat of fusion, and *T_m_* is the melting temperature.

In this note we focus on the kinetic escape pathways from metastability. We have observed the adiabatic freezing of water by fast imaging (8000 frames/s) in a liquid sample contained inside a 0.5 mm thick cell. The local temperature was synchronously monitored by a small K-thermocouple with a diameter of 75 *µ*m, a time resolution of 0.07 s, and a reading accuracy of 0.01 K. The sample was initially cooled down to a temperature *T*, upon equilibration with a thermostat. Then, the solidification was induced through a short (1 ns) laser pulse focused on the upper part of the sample cell (see the Supplementary Information for more details). It should be noted that at small-undercooling conditions (*T* > 267 K) the system is able to dissipate relatively large local perturbations, the energy of the pulse required to trigger the transition being relatively high (up to 100 mJ). At lower temperatures a pulse of about 15 mJ is strong enough to promote the transition. Sometimes we have even observed the spontaneous triggering of the phase transformation. Data for such cases have also been collected.

Figure 1 shows two frame sequences which display the process of ice growth at two different temperatures. The formation of solid dendrites on freezing is a quite general and well-known phenomenon^33^,^34^,^35^,^36^,^37^,^38^,^39^. Upon forming dendrites, the solidifying system does actually maximize the area of the solid-liquid interface, thus making the release of latent heat more efficient and, corrrespondingly, pushing the rate of entropy production to a maximum (see Ref. 40 and references contained therein). It has been suggested that the growth velocity of a dendrite is controlled by the rapidity with which heat diffuses away from the advancing crystal-melt interface^33^,^34^,^40^.

By visual inspection of frames like those exhibited in Fig. 1, we extracted an average growth velocity at various temperatures. However, before discussing these results we want to underscore a major difference between the present measurements and those performed by other authors (see, e.g., Refs. 33–39, 41, 42). In these papers the dendritic growth velocity was typically estimated by monitoring the velocity of propagation of a single dendrite tip. The corresponding data roughly conform to theoretical predictions^43^. Usually, the images show a single regular dendrite (relatively larger than the structures that we observed) which grows at a constant average velocity under steady-state conditions. On the contrary, we typically observed distorted dendritic branches which start growing on various (almost parallel) planes at different times and with different growth velocities. The situation is well illustrated in Fig. 1. In the upper panel two frames are shown, delayed by 0.03 s, which have been extracted from a movie recorded at *T* = 266.6 K. Three points were marked in the first frame, whose displacements are also reported in the second frame. Considering that the size and geometry of the cell force dendrites to grow along almost parallel planes, we could compute local growth velocities from the observed displacements, obtaining *v*_1_ = 0.71 cm/s, *v*_2_ = 0.61 cm/s, and *v*_3_ = 0.46 cm/s at the three chosen spots. The inspection of further frames from the same movie gave similar indications: we found some spread in the velocity values but growth velocities turned out to be, on the average, similar. Any measurement performed at temperatures lower than 266.6 K led to the same conclusion, except for the fact that the average growth velocity slightly increases on cooling. The sequence reported in the lower part of Fig. 1 was obtained for *T* = 267.9 K. Following the same procedure illustrated above, from the first two frames (delayed by 0.05 s) the estimated growth velocities at three different spots on the solidification front were *v*_1_ = 0.76 cm/s, *v*_2_ = 0.42 cm/s, and *v*_3_ = 0.69 cm/s. These values do not appreciably differ from those obtained for *T* = 266.6 K. The third frame in the bottom sequence of Fig. 1 is delayed by 0.1 s with respect to the second. The values of the average growth velocities obtained at the four marked spots were *v*_1_ = 0.23 cm/s, *v*_2_ = 0.26 cm/s, *v*_3_ = 0.28 cm/s, and *v*_4_ = 0.28 cm/s, respectively. The analysis of further frames taken from the same movie showed that the growth velocity gradually decreases with time. This behavior was observed for any measurement performed at temperatures higher than 266.7 K. We never observed a constant growth rate. Even monitoring the velocity of a single dendrite tip, we neatly detected a decrease of the velocity with time (note that two values out of those obtained from the bottom sequence reported in Fig. 1 refer to the tip velocity of the same dendrite at different times).

The discrepancies between our data and those found in the literature can be ascribed to some relevant differences in the experimental setups. Previously reported data were all obtained with sample cells that are modified versions of that originally proposed by Glicksman^44^. The apparatus typically consists of two distinct parts, viz., a nucleation cell and a larger crystal growth cell, connected through a glass capillary pipe (see, e.g., Fig. 2 in Ref. 36). Upon supercooling water contained in the nucleation cell, the solid phase eventually nucleates and starts growing, propagating through the capillary pipe into the second cell, whose temperature is set at a different undercooling level. A single dendrite grows in the second cell under the action of the given temperature gradient, from a macroscopic nucleus whose size is comparable with the section of the capillary pipe (see Fig. 4 of Ref. 36). Instead, in our experiment we tried to minimize all temperature gradients within the sampled volume. The laser shot was focused on the outer surface of the sample cell at a position which is about 1 cm far from the sampled volume. The transition was mainly promoted by the local energetic pulse and any heat possibly transferred by the laser had not enough time to propagate to the observation spot. Solidification started from a microscopic nucleus located at an arbitrary position within the sample and the dendritic structure then propagated throughout the sampled volume with a “random walk” dynamics. This picture was confirmed by the observation that no difference was ever detected between the processes occurring spontaneously and those triggered by the laser pulse.

The average growth velocities of dendrites located at various positions, estimated for different times in samples undercooled to different temperatures, are plotted in Fig. 2 in dimensionless units (*V* = *vd*_0_/(2*D*) *vs.* Δ = Δ*T* *C_p_*/*L*, where *v* is the measured velocity, *D* is the thermal diffusion coefficient, Δ*T* = *T_m_*−*T*, 
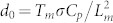
, and *σ* is the surface tension). The present data can be compared with those reported in the literature and with Langer's theoretical prediction^43^. It is apparent that, even if our data are affected by a larger spread, their trend matches fairly well with that of previous findings.

The time evolution of the temperature while the system is adiabatically freezing is plotted in Fig. 3. In this picture the time *t* = 0 marks the beginning of the phase transformation. Water freezing at very low temperatures reaches a stable equilibrium condition at *T* = *T_m_* in a very short time (Δ*t* < 0.1 s), whereas, for smaller undercoolings, a much longer relaxation time of a few seconds is needed. To our surprise, the crossover threshold between the *fast* and *slow* relaxation regimes is very sharp (266.6K < *T*_X_ < 266.7 K). In the slow-decay regime the time-dependent temperature can be fitted to 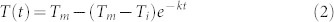
where *T_i_* is the temperature at *t* = 0 and *k* is a free parameter. It turns out that the whole set of data collected for *T_i_* > 266.6 K is fairly well reproduced with a unique value for *k* (1.3 s^−1^), independently of the initial temperature.

To our knowledge, such a sharp transition between two distinct kinetic regimes in the irreversible approach of metastable supercooled water to thermodynamic equilibrium has never been reported before. We also notice that the crossover threshold from a fast to a slow decay falls within the same narrow temperature range (marked by a vertical blue line in Fig. 2) where previously reported data for the dendritic growth velocity start deviating from the theoretical prediction^37^. It has been suggested that for moderately small supercoolings the growth velocity of a dendrite is affected by the coexistence of ice crystals with different morphologies^35^,^43^,^45^. It has also been observed that, far from coexistence, the natural convective motions of the liquid hosting the solid grains are enhanced with respect to what happens for smaller supercoolings^35^,^37^,^45^.

In order to determine how fast latent heat is transferred from a solidifying volume to the surrounding liquid in the slow-decay regime, we monitored the time evolution of the temperature under different experimental conditions. In particular, we performed measurements also in larger samples where the local temperature was simultaneously detected by two different thermocouples placed at a relative distance ranging between 8 and 15 mm (see the Supplementary Information for more details). We found no correlation between events occurring in different volumes of the same sample. In all cases the temperature happens to follow Eq. (2) with the same *T_i_* for each sensor. In addition, the time delay between the two solidification events is completely random, in agreement with a solidification front that propagates as a random walk. In both fast and slow-decay regimes the time dependence of the local temperature, as registered by each thermocouple, agreed well with the master plot shown in Fig. 3.

If we take the correlation between the average growth velocity of a dendrite and the temperature (see Fig. 2) seriously, we are led to conclude that the different growth velocities estimated through our movies provide a clear evidence that, as time goes on, the nucleation process occurs in the spatial region at contact with the dendrite surface at temperatures increasing progressively from *T_i_* to *T_m_*. The apparent contradiction between a solidification process taking place adiabatically on a macroscopic scale and the existence of local heat fluxes between the growing solid and its liquid neighborhood can be explained by noticing that the temperature gradients which are responsible for the heat fluxes are extinguished over distances that are definitely shorter than the cell size. For small supercoolings, when the growth process is slow, there is enough time for the establishement of heat fluxes and convective motions between the equilibrated liquid layer at *T_m_* and the colder bulk liquid, still at the initial temperature *T_i_*. The observation of two distinct time-evolution regimes of the sensor temperature can be ascribed to the temperature dependence of the balance of heat fluxes between locally coexisting phases. Below *T* = 267 K the growth process is faster while smaller volumes of water are warmed up. As a result, heat fluxes between the thin layers of water at *T* = *T_m_* do not appreciably propagate into bulk water at *T* = *T_i_*.

The formulation of a detailed theory behind the kinetic crossover at 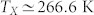 goes well beyond the aim of this note. However, a very simple model can give some support to the above picture. Let us disregard any detail about the dendrite geometry and fractal dimensionality and focus on the growth of a spherical ice embryo. With reference to Fig. 4, upon denoting as *v*(*T_i_*) the growth velocity of the solid grain which nucleates from the supercooled liquid at the initial temperature *T_i_*, the grain radius after a time Δ*t* from nucleation is *r*(*T_i_*) = *v*(*T_i_*)Δ*t* (assuming a vanishing critical radius). The solidification of a volume *V*(*T_i_*) = (4/3)*πr*(*T_i_*)^3^ of water in a time Δ*t* produces heat at a rate 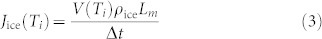
where *ρ*_ice_ is the mass density of the solid at *T* = *T_m_*. The volume of liquid in equilibrium with the solid at *T_m_* within the time Δ*t* can be calculated from Eq. (3). When the molar volumes of the coexisting liquid (*v_w_*) and solid (*v*_ice_) phases are known, it is straightforward to calculate the radius of the liquid layer surrounding the solid nucleus as 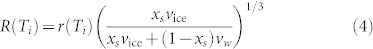
Looking at Fig. 5, the heat rate across the surface *S* = 4*πR*(*T_i_*)^2^ separating the kinetically coexisting volumes of liquid water at the temperatures *T_m_* and *T_i_* respectively, can be written as 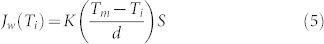
where *d* is the thickness of the layer separating the liquid phases at different temperatures and *K* = 0.58 Wm^−1^K^−1^ is the thermal conductivity of water.

Equation (5) requires an estimate of *d*, the thickness of the layer where the temperature gradient occurs. For temperatures falling in the fast-decay regime, large amounts of solid are generated in a short time and should be equilibrated with relatively small volumes of water. Hence, one can imagine that the heat transferred from the equilibrated liquid towards the colder liquid still at *T_i_* is lower than the released latent heat. At higher temperatures the situation is reversed. In absence of any other information, we use *d* as a free parameter and look for the value which fulfills the condition −*J_ice_*/*J_w_* = 1 at *T_X_*. We thus obtained 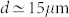, which looks as a reasonable guess. The resulting temperature behavior of the ratio between the two heat fluxes is reported in Fig. 5.

Another possible explanation for the existence of two different growth regimes may be traced back to the empirical observation that the stable heterogeneous state produced at the end of the adiabatic freezing process effectively resembles a colloidal gel of ice dendrites dispersed in liquid water, at least for initial temperatures falling in the fast-decay regime. In order to observe gelation, the volume fraction *φ* of the dispersed solid phase must overcome a threshold value. For structures grown in a diffusion-limited aggregation regime, numerical simulations indicate a fractal dimension of 1.5^46^,^47^, which agrees well with the experimental value for ice dendrites^37^. Small-angle light-scattering experiments on the colloidal gelation of fractal clusters with dimension 1.5^48^ have shown that a stable gel phase is first obtained for *φ* = 0.08 or slightly lower values. Equation (1) yields for the volume fraction of ice produced at the crossover temperature a value of about *φ* = 0.09. Hence, one can reasonably imagine that the convective motions of the liquid allowed at higher temperatures become suddenly hindered as, upon lowering the temperature, the percolation threshold is eventually reached and then overcome.

The present experimental results show that the decay of metastable water to equilibrium involves huge temperature fluctuations resulting in the ignition of heat fluxes in a bulk sample. As a result, different macroscopic phases, both solid and liquid, with different local temperatures coexist at sufficiently short times.

## Author Contributions

F.A. conceived the idea and wrote the manuscript. P.V.G. critically revised the thermodynamical argumentations and contributed to the detailed description of the observed irreversible process. R.C.P. performed the experiments and analyzed the data. S.P. developed the details of ice nucleation under adiabatic conditions. F.S. took care of the theoretical details and of the detailed comparison with previous literature data. G.S. designed the experimental set-up and wrote the acquisition software. C.V. supervised all the experimental work. All authors discussed the results and commented on the manuscript at all stages.

## Supplementary Material

Supplementary Information Supplementary Informations

## Figures and Tables

**Figure 1:**
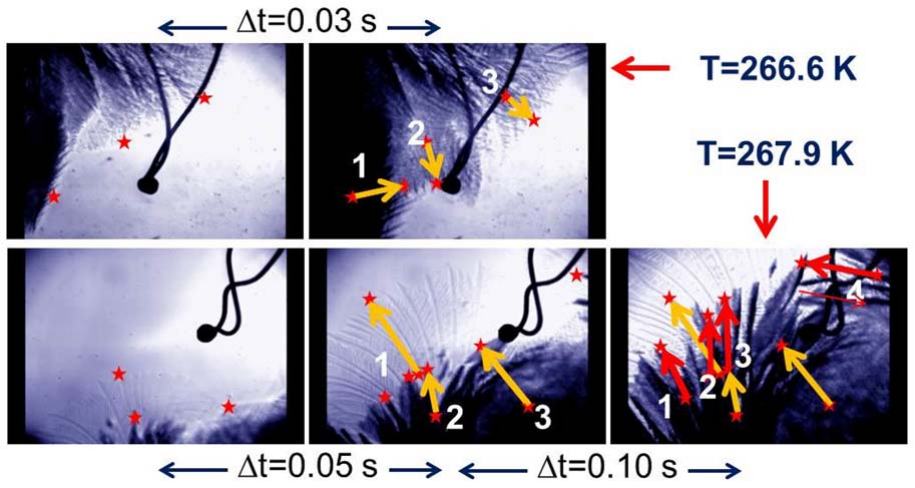
Two sequences of frames showing supercooled water freezing at two different temperatures (recording frame rate: 8000 fr/s; spatial resolution: 1.1 *µ*m/px; field of view: 1.1 × 0.7 mm^2^). The time delay between two adjacent frames in each sequence is indicated. The thermocouple which monitors the local temperature is visible at the center of each image.

**Figure 2:**
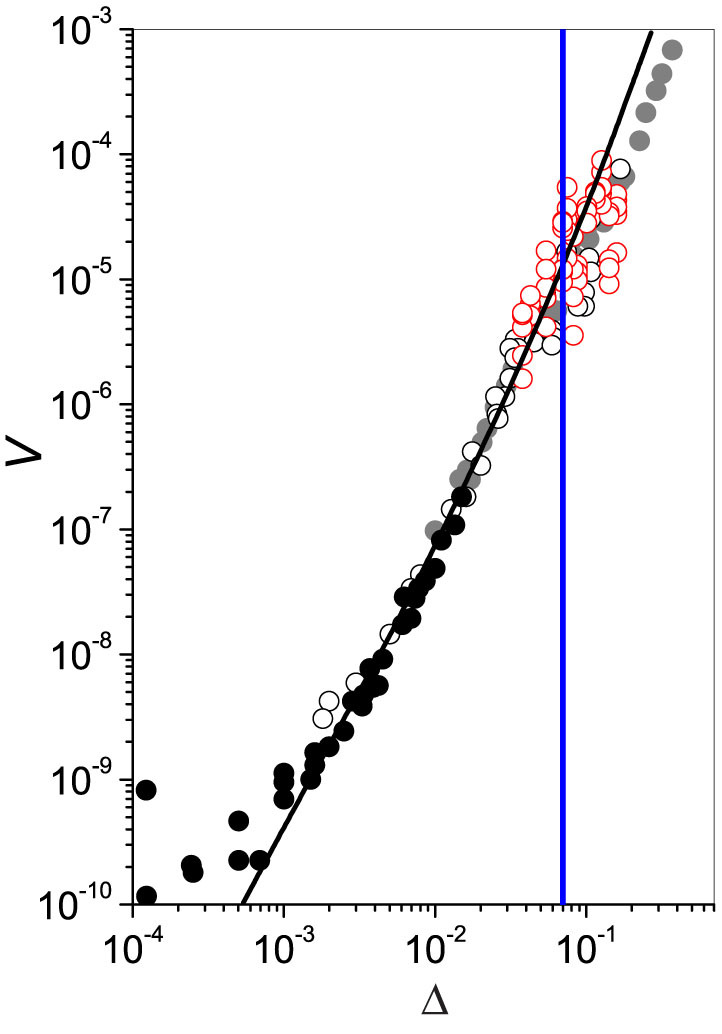
Growth velocity plotted as a function of supercooling (see the main text for the definition of both dimensionless quantities): black symbols refer to data taken from Ref. 37 while the present data are shown as open red circles. The continuous black line represents the theoretical prediction given in Ref. 43. The vertical blue line marks the temperature of the observed transition.

**Figure 3:**
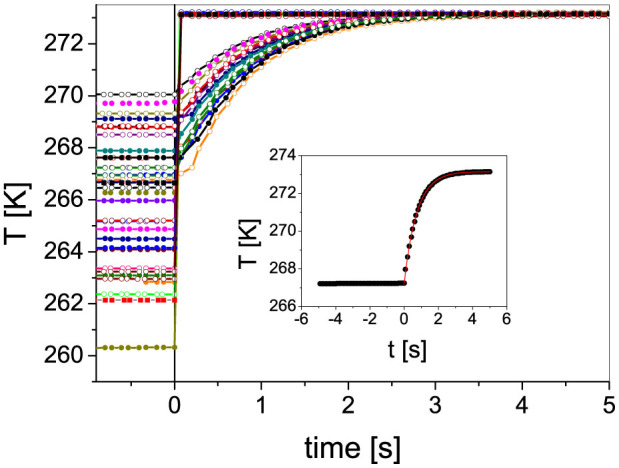
Time evolution of the local temperature in supercooled water during freezing. The time *t* = 0 marks the beginning of the process (triggering). We report in the inset the time behavior of the temperature, measured in water originally undercooled to *T_i_* = 267.22 K, together with the result of a fit of the data carried out with Eq. (2).

**Figure 4:**
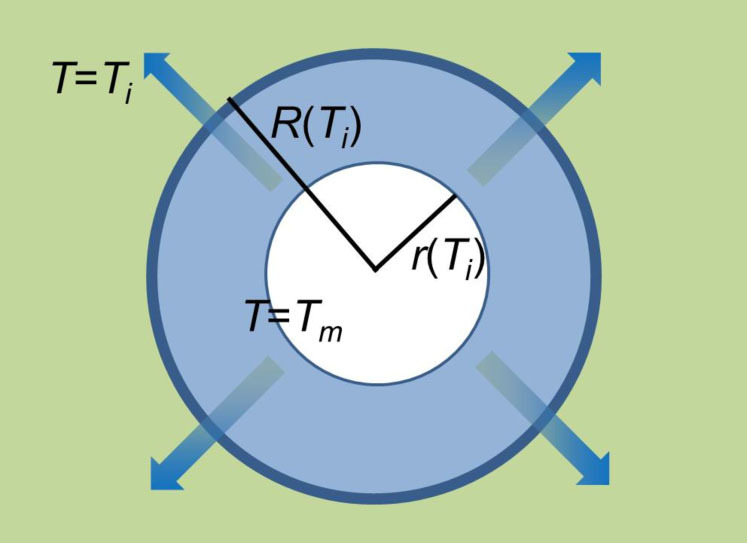
Model scheme of the growth process of an isotropic solid nucleus in a time Δ*t*: the white sphere at the center of the image represents the growing solid phase at equilibrium - i.e., at the coexistence temperature - with the surrounding layer of water (cyanide corona). The outer green area represents the bulk water which has not been reached yet by the propagating fluctuations, still at the initial supercooling temperature *T_m_*. The blue band separating the liquid volumes at different temperatures represents the liquid layer over which the temperature gradient is established (see text for details).

**Figure 5:**
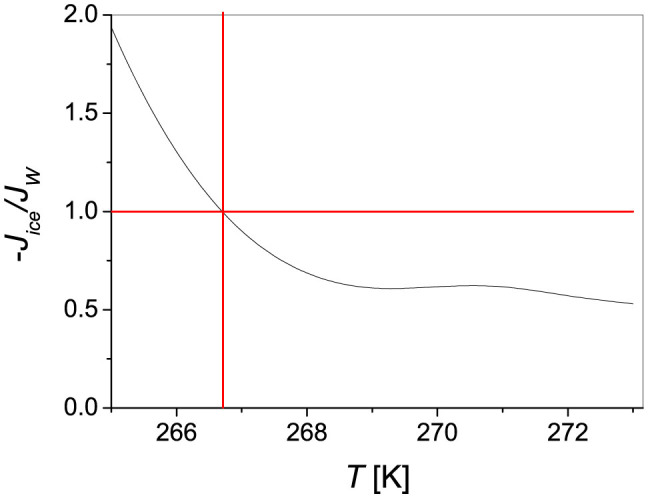
Temperature dependence of the heat fluxes towards and from the liquid water volume at equilibrium with the growing solid phase (see text for details).
