# Supplementary material for: Supercooled water escaping from metastability
Source: Sci Rep. 2014 Nov 27;4:7230. doi: 10.1038/srep07230 (PMC4245691; doi:10.1038/srep07230)
Supplement: Supplementary Information — s [file srep07230-s1.pdf]

Supercooled water escaping from metastability

Francesco Aliotta[1], Paolo V. Giaquinta[2], Rosina C. Ponterio[1],

Santi Prestipino[1,2], Franz Saija[1], Gabriele Salvato[1], and Cirino Vasi[1]

[1] *CNR-Istituto per i Processi Chimico-Fisici,*

Viale Ferdinando Stagno d'Alcontres 37, 98158 Messina, Italy

[2] *Università degli Studi di Messina,*

Dipartimento di Fisica e di Scienze della Terra,

Contrada Papardo, 98166 Messina, Italy

Supplementary Information

A. Validity of the adiabatic approximation

We show here that, under any accessible experimental condition for bulk samples, the return of supercooled water towards stable equilibrium takes place adiabatically. To this aim, we compared the enthalpy content of a supercooled sample with that of the same sample immediately after adiabatic-freezing completion. The validity of the adiabatic approximation was tested by calorimetric measurements. A 10 cc vial was cooled down to different temperatures, both in the stable and metastable regimes. After thermalization, the vial was transferred into a calorimeter and the heat exchange required for thermal equilibration with the calorimeter bath was measured. The same procedure was repeated after cooling down the same vial to the same temperature, but promoting the transition through a mechanical shock before inserting the vial into the calorimeter. The results are reported in Fig. 1: both sets of data approximately lie on a straight line, which demonstrates that the adiabatic approximation can be taken safely. In the same figure we also report data from two measurements performed on systems which, after promoting the transition, were allowed to equilibrate with the thermostatic bath. Their positions in the plot give a good indication of the measurement accuracy. The measurements ensure us that, within the experimental uncertainty, the transition towards stable equilibrium takes place over a shorter time than that required for the establishment of local heat fluxes between the sample and its environment. Since the measurements were performed on a sealed vial and adiabatic freezing implies a volume change, our experiment was not carried out at constant pressure. However, the phase diagram of water, with a liquid-solid coexistence temperature that is almost insensitive to any pressure change around normal conditions, ensures us that any moderate variation of the pressure occurring during the process does not significantly affect the stable state towards which the system evolves. It is also easy to see that the moderate pressure change occurring in the system plays a negligible role on the observed volume change. In this perspective, the work associated with the pressure change on the gas phase in the vial can be safely neglected. Hence, we conclude that the process involving the condensed phases of water inside the vial can be described within the adiabatic approximation.

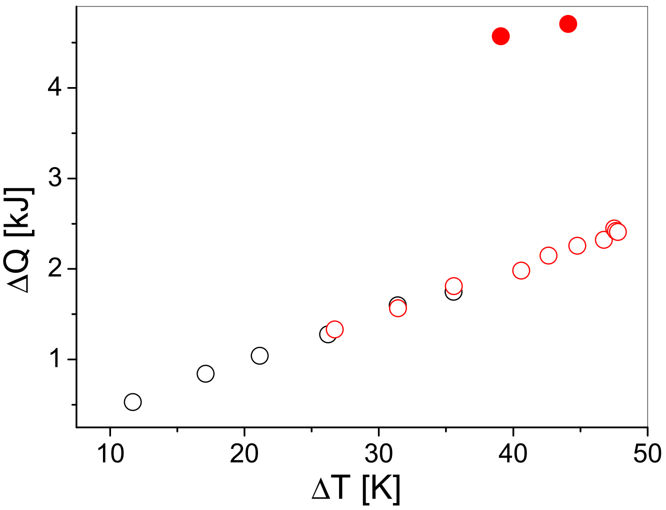

FIG. 1: Calorimetric measurements of the heat exchanged between a 10 cc vial of water and the calorimeter bath. The data are plotted as a function of the calorimeter temperature change, ΔT. Open black circles are data collected from liquid samples (both stable and metastable) cooled down to different temperatures. Open red circles are data from supercooled samples in which the transition to the stable phase has been promoted before the measurement. Full red circles are data from equilibrated systems which were maintained in the thermostatic bath in order to reach thermal equilibration before the calorimetric measurement.

B. Experimental details

The sample was put in a fused quartz cell with dimensions 9.3 mm×38.9 mm×0.5 mm. We used an optical thermostat which consists of a flat metallic finger facing one of the larger cell faces for the whole cell height (see Fig. 2). A hole at the center of the finger allowed us to observe the sample. The finger is monolithic with a large bottom plate that was cooled by a cascade of two Peltiers couples. The body of the cell is made of ertalon and an o-ring seal is interposed between the base plate and the body of the thermostat. Two fused quartz windows (5 mm thick) allowed the observation of the sample and lighting (in transmission). On the sides of the two windows two small holes fit two cannules through which nitrogen flows to prevent humidity condensation. Ethyl alcohol was used as cooling bath. There was no warm source because thermal exchange with the environment turned out to be sufficient. The temperature of the sample in the observed volume was sampled through a tiny K

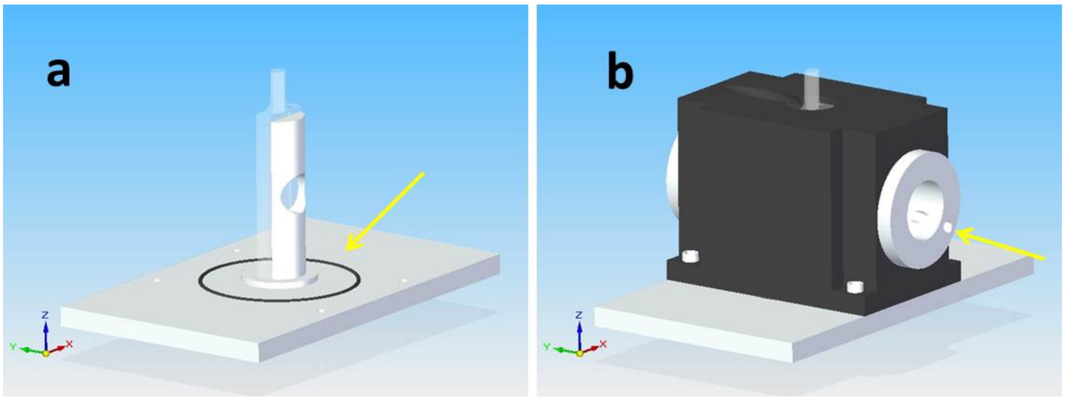

FIG. 2: Design of the optical thermostat. **a**: cooling finger with base plate and the sample cell in working position; the arrow indicates the o-ring seal; **b**: assembled thermostat. The arrow indicates the entrance of the channel though which nitrogen flows so as to prevent humidity condensation.

thermocouple with a diameter of 75 μm. The thermostatation accuracy in the sampled volume was better than 0.01 K, in the range 243 K $\leq T \leq$ 273.15 K. The measurements were performed in the temperature range 260.3 K $\leq T \leq$ 270.1 K. The transition of the metastable sample was promoted by a laser (Yag-Nd, Brilliant Eazy) pulse (1 ns, λ=532 nm) focused on the top of the cell (1 cm far from the sampled volume). In some occasions, particularly at the lower explored temperatures, the transition occurred spontaneously. Data from spontaneous transitions were also collected. High speed (8000 frames/s) were collected with a Photron Fastcam SA4 camera. The signal from the thermocuple was monitored on line after analog-to-digital conversion by a Lakeshore 331 unit (time resolution 0.07 s). The measurements were performed following the following procedure:

i) The sample was stabilized at the preset temperature.

ii) The camera started collecting frames on a circular buffer (size 4096 frames).

iii) The temperature readings were collected on a circular buffer (size 143 elements) by the online computer.

iv) An area of 10 px$\times$10px at the center of the images was continuously monitored; when the integrated intensity from this area changed by 1%, we assumed that the transition was occurring. The camera generated an internal trigger signal and the image aquisition proceeded for half of the buffer size and then stopped. Thus, a sequence was stored in which the central frame was the one collected at the trigger time.

v) The trigger signal was sent to the computer. The temperature readings proceeded for

half of the buffer size; in such a way the central element of the stored array was synchronous with the central frame of the movie.

Some test measurements of the temperature evolution during the transition were repeated in the larger samples (10 cc and 1000 cc), monitoring the local temperatures at two different sample volumes (the distance between the sensors ranged between 8 mm and 15 mm) by two thermocouples. These samples were thermostated by inserting them inside a thermostatic bath (accuracy ≈ 0.1 K, reading accuracy ≈ 0.01 K). The transition was promoted by a drop of liquid nitrogen falling on the top of the sample container (10 cc sample) or by a mechanical shock (1000 cc samples). Readings from spontaneous transitions were also collected. These measurements were performed over the temperature range 266 K $\leq T \leq$ 270 K.
